# Minimally invasive transiliac anatomical locking plate for posterior pelvic ring injury: a retrospective analysis of clinical outcomes and radiographic parameters for the gull wing plate

**DOI:** 10.1186/s12891-022-05829-1

**Published:** 2022-09-22

**Authors:** Sadaki Mitsuzawa, Kenji Kusakabe, Shota Nakao, Tetsuya Matsuoka, Tadashi Yasuda, Shuichi Matsuda

**Affiliations:** 1 Department of Orthopaedic Surgery, Kobe City Medical Center General Hospital, 2-1-1 Minatojima-minamimachi, Chuo-ku, Kobe, 650-0047 Japan; 2 Department of Orthopaedic Trauma, Senshu Trauma and Critical Care Center, Rinku General Medical Center, Osaka, Japan; 3 Department of Orthopaedic Surgery, Kyoto University Graduate School of Medicine, Kyoto, Japan

**Keywords:** Pelvic ring injury, Sacrum, Sacroiliac joint, Posterior fixation, Transiliac plate

## Abstract

**Background:**

Posterior pelvic ring injuries are challenging for surgeons to treat adequately due to difficulties with reduction and stabilization. Surgical intervention is a beneficial option to protect neurological structures and provide sufficient stability for early mobilization. The gull wing plate (GWP) is a pre-contoured anatomical locking plate with six screws, and its design is unique among posterior transiliac tension-band plates. The purpose of this study was to investigate clinical results of the GWP.

**Methods:**

Patients who had an unstable posterior pelvic ring injury and underwent internal fixation with GWP were retrospectively analyzed at a trauma center. Demographic data, fracture type, perioperative data, and radiological evaluation with computed tomography (CT) were collected. Clinical outcomes were graded using the functional independence measure (FIM) and Majeed outcome score.

**Results:**

Twenty-six patients were enrolled (mean age, 54 years), and the mean follow-up period was 23 months. The mean Injury Severity Score was 24 points, and internal fixation was performed 6.6 days post-trauma. CT evaluation showed the lateral surface angle of the uninjured ilium was approximately 68°. The GWP pre-contoured anatomical design closely matched this angle. The mean FIM and Majeed score were 119 and 76 points, respectively, which were graded as excellent (*n* = 14), good (*n* = 9), or fair (*n* = 3).

**Conclusions:**

With the retrospective single-center data available, the GWP seems to be a minimally-invasive alternative, provides reliable stability of the posterior pelvic ring and allows for rehabilitation within normal ranges.

## Background

Posterior pelvic ring disruptions are commonly caused by sacral fractures or iliosacral joint dislocation. To avoid long-term bedrest and complications, surgical intervention is critical to achieve sufficient stability for early mobilization [1]. However, these injuries are still challenging for surgeons to treat adequately due to difficulty with reduction and stabilization. Various methods for internal fixation have been reported, such as percutaneous iliosacral screws, transiliac bars, spinopelvic instrumentation, sacral plates, or transiliac plates [2].

**Fig. 1:**
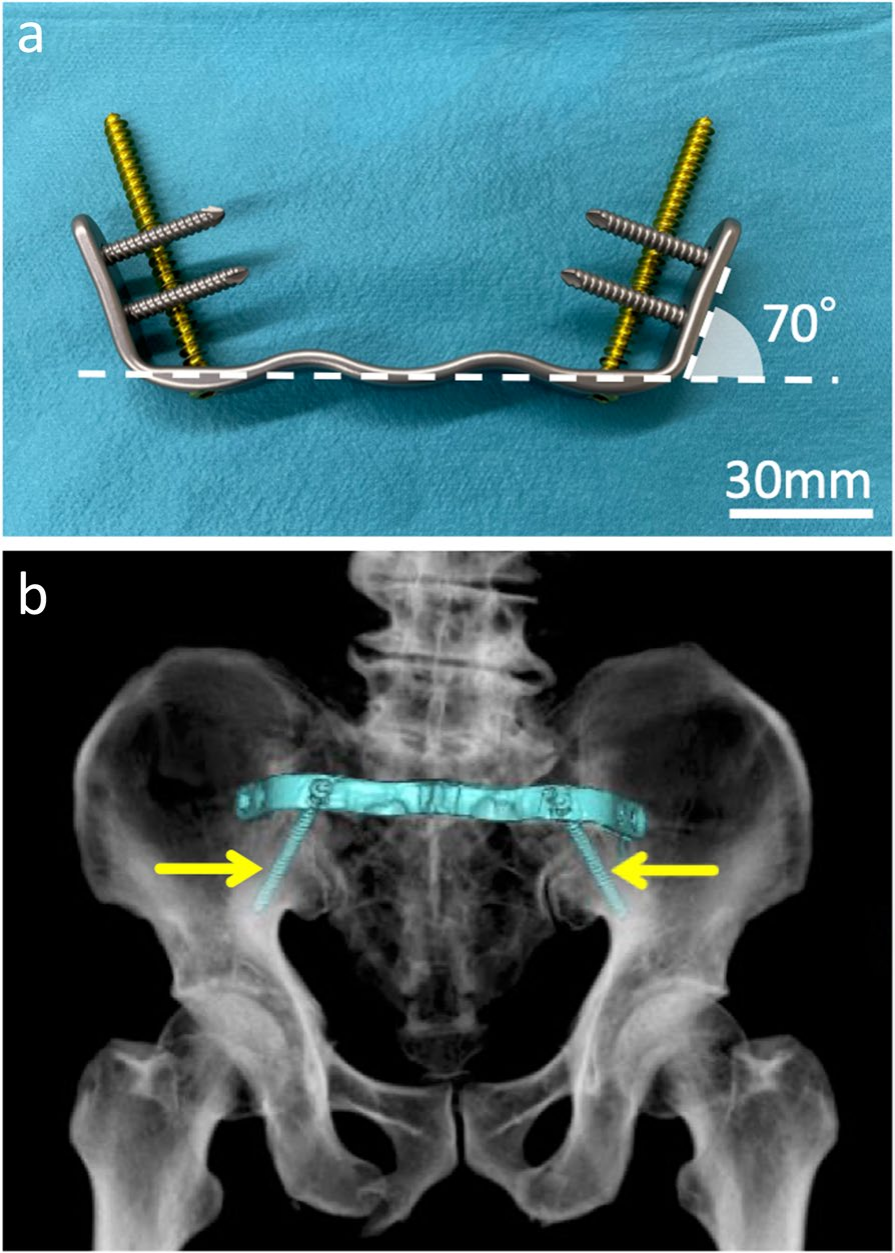
Characteristics of the gull wing plate. **a** The GWP design takes into account the bilateral ilia, S1 spinal process, and paravertebral muscle. The angle between the posterior surface and the bilateral wings is 70°. Two longer cancellous screws (φ6.5 mm; gold) toward the AIIS and four tri-cortical locking screws (φ5.0 mm; silver) penetrating the iliosacral joint can be inserted. Scale bar: 30 mm. **b** 3D reconstruction of the postoperative CT scan from the posterior side. Bilateral cancellous bone screws are inserted as far as possible (yellow arrows). GWP, gull wing plate; AIIS, anterior inferior iliac spine; CT, computed tomography

Percutaneous iliosacral screws seem to be the least invasive and the most promising method. However, the surgery is technically demanding, and complications such as iatrogenic neurovascular injury and postoperative screw loosening cannot be overlooked [3–6]. Minimally invasive transiliac tension-band plates may be an alternative because of their safety and mechanical stability. The gull wing plate (GWP; OMIC Corporation, Shiga, Japan) is a pre-contoured anatomical locking plate with two cancellous screws (φ6.5 mm) and four locking screws (φ5.0 mm), and it is widely accepted and used as a posterior transiliac plate in Japan (Fig. 1). Clinical outcomes of posterior transiliac plates with a similar design have not been reported. The purpose of this study was to investigate our results using the GWP.

## Methods

The study protocol and research were performed in accordance with the Ethics Committee at our institution. Between July 2018 and August 2021, patients who had a posterior pelvic ring injury and underwent GWP were enrolled into this retrospective study. Patients who required posterior spinal instrumentation for vertically displaced or bilateral severe sacroiliac joint injuries were excluded.

### Surgical technique and postoperative protocol

Internal fixation with the GWP was performed as reported precisely in the previous study [7]. Briefly, before surgery, the optimal plate size can be planned with reference to the distance between bilateral posterior superior iliac spine (PSIS) on the axial computed tomography (CT) slice. Fixation of an unstable anterior pelvic ring is performed in the supine position, if necessary. In the prone position, approximately 5-cm longitudinal incisions are designed at the bilateral PSIS at the level of the first sacral vertebra (S1). After creating a 1.5 cm × 1.5 cm bone groove at the top of the PSIS, the paravertebral muscle (PVM) is elevated from the posterior surface of S1, and the spinal process is resected to create a tunnel connecting the bilateral incision. If reduction for a rotation deformity is necessary, a Schanz pin can be inserted from the PSIS, and the displaced ilia can be reduced by tilting that pin medially or laterally using the joy stick technique. For reducing a vertical deformity, a Schanz screw at the femoral neck or Kirschner wire direct traction from the distal femoral can be useful. The selected plate is inserted beneath the PVM, and bilateral cancellous bone screws (φ6.5 mm) are inserted as far as possible towards the anterior inferior iliac spine (AIIS). After tri-cortical drilling through locking screw sleeves at the plate wings, locking screws (φ5.0 mm) of a selected length are inserted using the torque lenti driver. The incised common fascia is completely repaired to cover the plate and reduce postoperative tenderness. For the postoperative rehabilitation protocol, all patients were mobilized under physiotherapeutic supervision with half weight bearing from post-operative days (POD) 1 to 13 and full weight bearing after 14 POD as tolerated.

### Case example

**Fig. 2:**
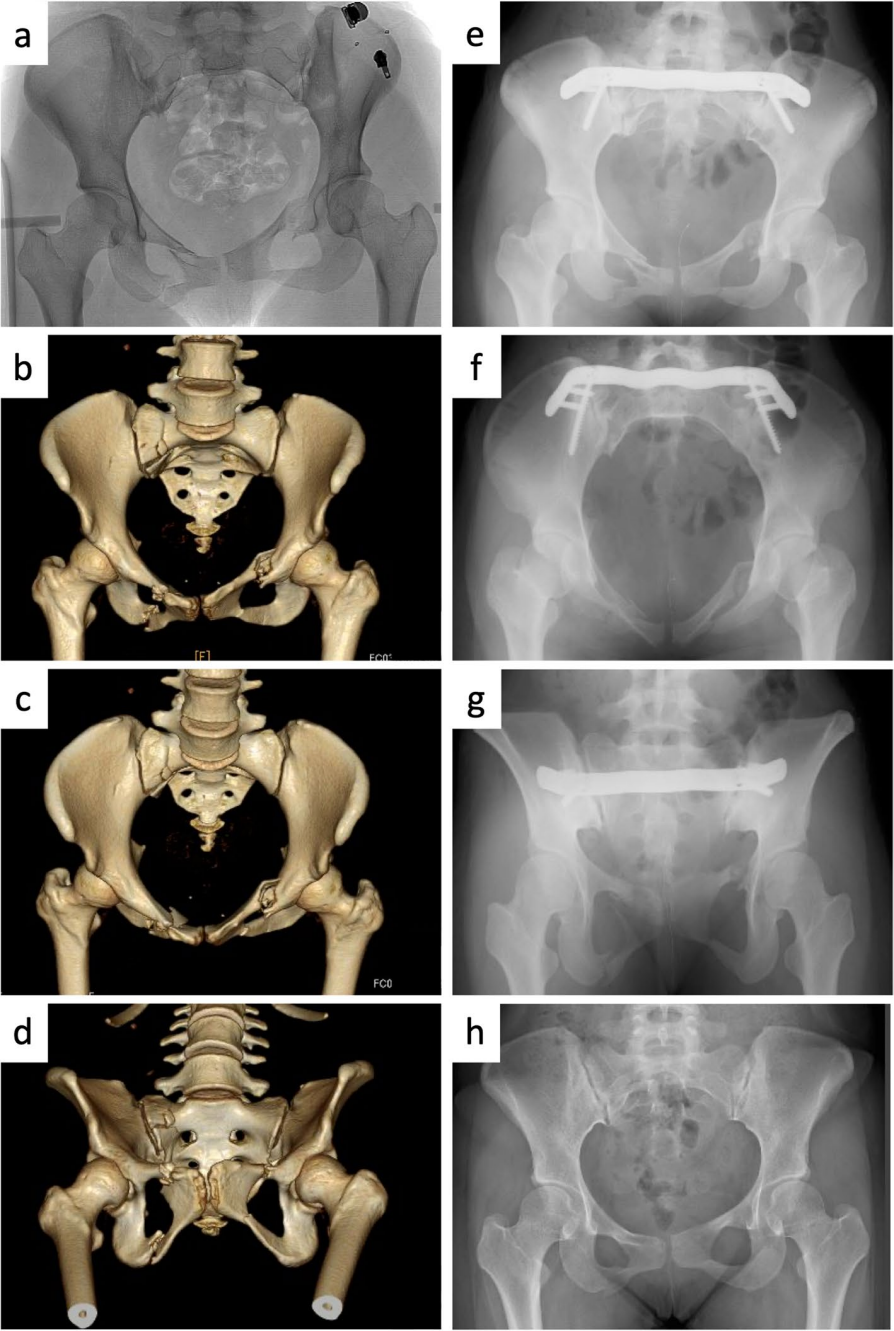
Case example. **a** Plain AP radiograph at the emergency room. **b** 3D reconstruction of the CT scan (AP view). **c** 3D reconstruction of the CT scan (inlet view). **d** 3D reconstruction of the CT scan (outlet view). **e** Postoperative radiograph (AP view). **f** Postoperative radiograph (inlet view). **g** Postoperative radiograph (outlet view). **h** Plain AP radiograph at 26 months postoperatively (after implant removal)

A female in her twenties was involved in a motor vehicle accident (Fig. 2). The patient had a pelvic ring injury (AO/OTA 61B2), right sacral fracture (Denis zone I), and bilateral superior and inferior pubic rami. She was initially managed with transcatheter arterial embolization (TAE) and pelvic external fixator at iliac crests. Internal fixation with a gull wing plate was performed on post-accident day 3. The operation time was 80 min, with an intraoperative blood loss of 20 ml. The patient had an excellent pelvic outcome (Majeed score: 87 points) at 35 months follow-up.

### Data collection

Patient demographic data included the following: age, gender, trauma mechanism, Injury Severity Score (ISS), associated lesion other than pelvis, AO/OTA classification, information about the ramus and sacral fracture (Denis classification) [8], preoperative neurological deficit (Gibbon classification) [9], the presence of the preoperative external fixator and TAE, trauma-to-surgery time, admission period, and follow-up period.

### Clinical outcome measurement

The following intraoperative information was recorded: surgical time (only for GWP, excluding fixation for anterior component), blood loss, plate size, iliac screw length, and complementary fixation for anterior component. Functional independence measure (FIM) at admission, discharge, and final follow-up were evaluated [10]. Majeed score at the final follow-up was recorded [11]. Postoperative complications and implant removal were investigated.

### Radiographic parameters

**Fig. 3:**
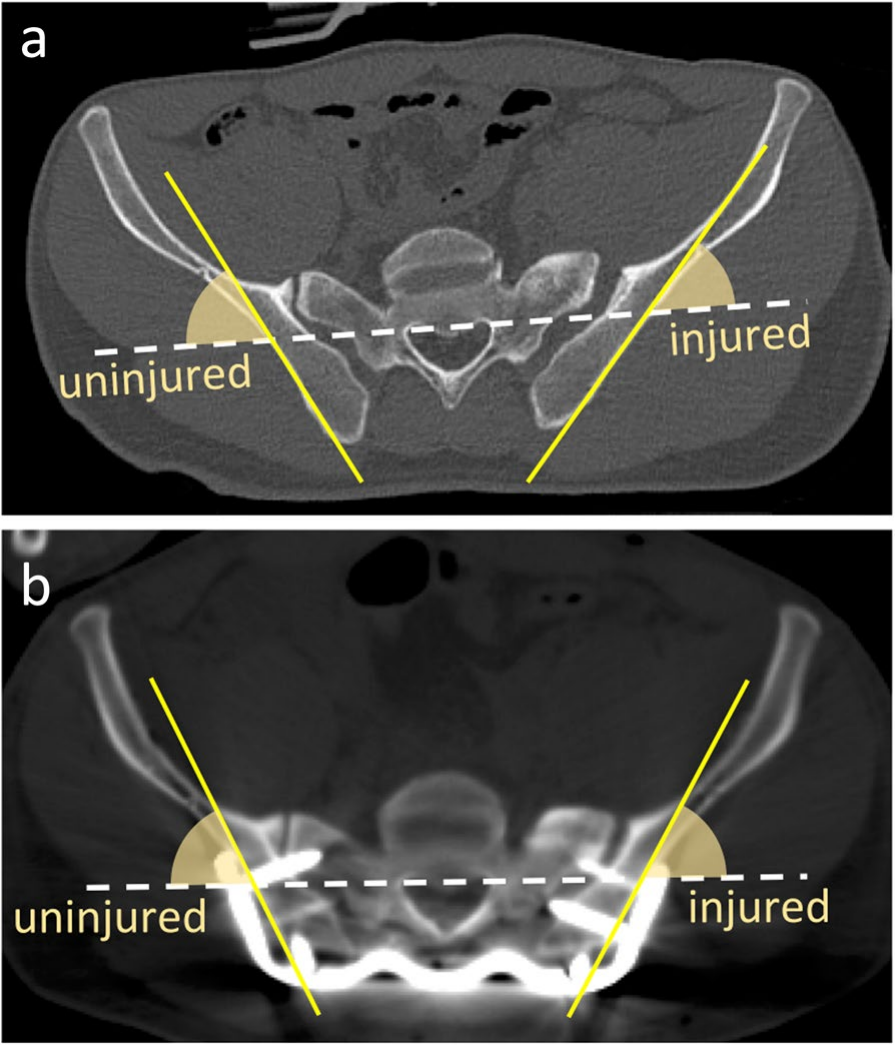
**a** Pre- and **b** Post-operative CT evaluation. The angle between the horizontal plane of the sacrum (white dotted line) and the lateral surface of the ilium (yellow line) were measured bilaterally

Pre- and post- operative CT evaluation (Fig. 3) was performed to ensure that the wings of GWP coincided with the lateral surface of the ilium. The axial slice where the S1 foramen ends at the anterior surface of the sacrum was selected. The horizontal plane of the sacrum was defined on the basis of the spinal canal and neural foramina (white dotted line). The lateral surface of the ilium was defined at the deep portion of the PSIS (yellow line). The angle between both lines was measured bilaterally. Next, the absolute value of difference between lateral surface angles of injured and uninjured ilium were calculated. Pre- and post-operative values were compared using the Student’s *t*-test. Values of *p* < 0.05 were considered to be statistically significant.

## Results

During the study period, 26 patients underwent GWP for posterior pelvic ring injury. Patient demographics are shown in Table 1. Eighteen men and eight women were enrolled, with a mean age of 54 years. The mean follow-up period was 23 months. The most common mechanism of injury was a traffic accident in 16 patients. The mean ISS was 24 points. The AO/OTA classification showed the following results: B1, six patients; B2, nine patients; B3, four patients; C1, two patients; and C2, five patients. Eighteen patients had a displaced anterior arch. Sacral fractures were classified as follows: Denis 1, six patients; Denis 2, 14 patients; and Denis 3, three patients. Although neurological deficits were recorded at the initial examination in six patients, further assessment was impossible in four patients due to other injuries, such as cervical cord injury, direct blow to the common peroneal nerve, and sciatic nerve injury followed by displaced distal femur fracture. Almost 70% of patients underwent external fixator and TAE on the day of injury. The mean timing of surgery was 6.6 days after trauma. The mean admission period was 34 days.

**Table 1:** Patient demographics

	(*n* = 26)
Mean age (years)	54 ± 22
Male: Female	18: 8
Trauma Mechanism	
Traffic accident	16
Fall from height	8
Pincer damage	2
ISS score	24 ± 11
Associated lesion	
Spinal fracture (Spinal cord injury)	15 (2)
Upper-limb fracture	13
Lower-limb fracture	8
Morel-Lavellee	0
Urogenital	2
Abdominal	5
Thoracic	15
Craniofascial	9
Heart and aortic	2
Traumatic CPA	2
AO/OTA classification	
B1	6
B2	9
B3	4
C1	2
C2	5
C3	0
Ramus fracture	
None	1
Nondisplaced anterior arch	7
Displaced anterior arch	18
Sacral fracture	
None	3
Denis Zone 1	6
Denis Zone 2	14
Denis Zone 3	3
Preoperative nuerological deficit	
Impossible to evaluate due to other injury	4
Gibbon 1	20
Gibbon 2	0
Gibbon 3	1
Gibbon 4	1
Preoperative external fixater	18
Preoperative TAE	19
Trauma-to-surgery time (days)	6.6 ± 3.3
Admission period (days)	34 ± 21
Follow-up period (months)	23 ± 13

*ISS* Injury Severity Score, *CPA* Cardiopulmonary Arrest, *AO/OTA* AO Foundation/Orthopaedic Trauma Association, *TAE* Transcatheter Arterial Embolization

**Fig. 4:**
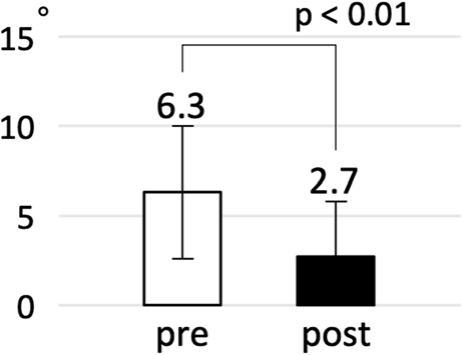
The absolute value of difference between lateral surface angles of injured and uninjured ilium. Post-operative values were demonstrated to be smaller than pre-operative values (*p* < 0.01)

Clinical results are shown in Table 2. The mean surgical time was 92 min, and the mean blood loss was 162 mL. The mid-sized (115 mm) GWP was most commonly selected (16 patients). The mean iliac screw length was 72 mm. Nine patients underwent fixation of the anterior pelvic ring before GWP. CT evaluation showed that the lateral surface angle of the uninjured ilium was approximately 68°, while that of the injured ilium was 65.9° ± 6.9° preoperatively and 67.9° ± 4.5° postoperatively. In terms of the absolute value of difference between injured and uninjured ilium, post-operative values (2.7° ± 3.1°) were significantly smaller than pre-operative values (6.3° ± 3.7°) (*p* < 0.01) (Fig. 4). No infections were recorded. One locking screw came out probably due to interference with the iliac screw. In accordance with the patients’ wishes, implant removal was performed in nine cases. The FIM improved over time, and it was 119 points at the final follow-up. The mean Majeed score at the final follow-up was 76 points, which were graded as excellent in 14 patients, good in nine patients, and fair in three patients. All three patients who were graded as fair, had associated spinal cord injury or neurological deficits and their symptoms were treated with medication or a prosthetic appliance.

**Table 2:** Clinical results

	(*n* = 26)
Surgical time (minutes)	92 ± 18
Blood loss (ml)	162 ± 133
Plate size	
Small (105 mm)	7
Middle (115 mm)	16
Large (125 mm))	2
Extra-large (135 mm)	1
Iliac screw length (mm)	72 ± 11
Complementary fixation	
Anterior plating	5
Superior ramus screw	3
LC2 screw from AIIS	1
Preoperative CT evaluation (injured / uninjured)	65.9 ± 6.9 / 68.1 ± 4.5
Postoperative CT evaluation (injured / uninjured)	67.9 ± 4.5 / 68.0 ± 4.4
FIM at admission	33 ± 18
FIM at discharge	82 ± 28
FIM at final FU	119 ± 10
Majeed score at final FU	76 ± 14
Excellent	14
Good	9
Fair	3
Poor	0
Complications	
Implant trouble (screw back out)	1
Infection	0
DVT	5
Hardware removal	9

*LC2* Lateral Compression Type2, *AIIS* Anterior Inferior Iliac Spine, *CT* Computed Tomography, *FIM* Functional Independence Measure, *FU* Follow up, *DVT* Deep Vein Thrombosis

## Discussion

Management of a pelvic ring injury poses significant challenges for orthopaedic surgeons [12]. An initial X-ray or CT scan in the emergency department might not reflect the potential displacement or instability of the injured pelvic ring due to the recoil capacity of the pelvis [13]. The posterior part of the pelvic ring is the key structure in terms of hemorrhage control and load transmission from the lower limbs to the lumbar spine. The sacrum forms joints with the bilateral ilia and contains the central canal and neural foramina. Because of its thin cortex, sacral fracture is often comminuted, and this often causes neurological deficits. Perfect reduction and rigid fixation, which provide sufficient stability for early mobilization, are essential to improve the patient’s quality of life.

Among several fixation methods, the percutaneous iliosacral screw or trans-iliac screw is the first choice in most cases. These screws can be used only for a minimal displacement, but a short surgical time, less blood loss, and lower risk of soft tissue complications are attractive features. However, there are some concerns related with percutaneous screw fixation. First, as reported in most studies, it requires extensive expertise, thorough knowledge of anatomical structure around the pelvis, and careful recognition of dysmorphic sacrum. Screw malpositioning, which directly leads to serious neurologic and vascular injury, should be avoided. Matiyahu et al. demonstrated that the rate of dysmorphism affecting the iliosacral screw trajectory was 17% in their randomized multicenter study [14]. Thus, with conventional fluoroscopy methods, malposition occurred in 12% (9/73) of screws in the normal sacral group and in up to 32% (7/22) of screws in the dysmorphic sacral group [14]. Sagi et al. presented a case report involving screw malpositioning that was apparently an appropriate position on intraoperative fluoroscopy [4]. They emphasized awareness of the tangential or oblique nature of the sacral foramina and concluded that the screw should be placed in the anterior portion and not just above the foramen in the outlet view [4]. Zwingmann et al. performed a systematic review and meta-analysis, and they estimated rate of malposition to be 2.6% (1832 screws) using the conventional fluoroscopy method, and this was significantly higher than using the CT navigation method, which had a malposition rate of 0.1% (262 screws) [6]. Therefore, recent studies recommended an intraoperative CT scan or a navigation system for optimal screw positioning [6, 14]. A second concern is related to percutaneous screw fixation and its biomechanical stability and postoperative implant difficulties. A biomechanical study revealed that bilateral iliosacral screws (8 mm in diameter) were inferior to the posterior tension band plate (4.5 mm, non-locking) [15]. The rate of implant failure, such as screw loosening or loss of reduction, was 7%–13% in clinical studies [3, 5]. Kim et al. demonstrated in their systematic review and meta-analysis that implant loosening was more common in the percutaneous screw group than in the posterior plate group [16]. Third, intraoperative radiation exposure for the percutaneous screw is higher than for other surgical methods. For accurate and safe positioning of the iliosacral screw or transiliac screw, the iliosacral screw was demonstrated to require 7.6-times more radiation exposure during surgery than that of the posterior transiliac plates [17].

Currently, several researchers have re-evaluated and verified the effectiveness and safety of posterior transiliac plates [18–22]. Among them, large-sized plates (4.5 mm) are preferred to small-sized plates (3.5 mm), and locking compression plates seem to have replaced conventional non-locking plates [22]. We suggest that the GWP three-dimensional design provides even greater stability compared with that of other plates because two longer cancellous screws (φ6.5 mm) toward the AIIS pass through a corridor with a high bone density and four tri-cortical angular-stable screws (φ5.0 mm) fix the iliosacral joint. The wing angle is 70°, which closely matches the angle of the uninjured positional relationship between the sacrum and ilium (approximately 68°) in the current study. The pre-contoured anatomical design is beneficial because bending the thick straight plates during surgery takes time, reduces the plate strength, and breaks the screw hole locking mechanism. While Ayoub et al. and Suzuki et al. reported a relatively low rate of infection of 7.5% (3/42 cases) and 10.5% (2/19 cases), respectively, there were no infections in our study cohort [18, 19]. The GWP was slipped beneath the PVM through bilateral longitudinal 5 cm incisions, and it was only minimal invasive in the soft tissue. The wavelike form of GWP is also gentle on the PVM, PSIS, and the S1 spinal process, which is partially resected by the osteotome. Compared with the open method from a midline incision, the shorter surgical time and reduced blood loss might contribute to the low rate of postoperative infection. However, before selecting GWP, soft tissue conditions such as a degloving injury should be carefully investigated. Another advantage of GWP is less radiation exposure. Because image intensifier is necessary only when checking the trajectory of iliac screw and the tip of the locking screw, a radiolucent fracture table is not required for GWP. Direct accessibility to the posterior surface of the sacrum is also a benefit of GWP. Autologous bone grafting using the resected PSIS can be easily applied to a sacral fracture site. When nerve decompression is required, a small, medialized skin incision enables a sacral laminectomy. However, in this case, another stab incision is necessary to set the locking screw sleeve. GWP clinical outcomes are satisfactory, and equal to or better than previous studies. FIM in the current study improved over time after admission. Near full activities of daily living (FIM, 119 points) was achieved at the final follow-up visit. Our patient cohort showed a Majeed score of 76 points and 88.5% had a satisfactory (excellent and good) result, while previous studies showed 71.8–78.5 points and 72.2%–80.0% with satisfactory results [18, 20, 21]. The achieved rigidity of GWP provides sufficient stability to allow early mobilization, weightbearing, and activities of daily living. There are only two contraindications for using GWP. The first contraindication is the fracture type, such as cranially/caudally displaced fractures and bilateral severe injuries around the sacroiliac joints, which should be suitable for fixation using spinal instruments that place anchors at the lumbar spine. The second contraindication is severely damaged soft tissue, such as a Morel–Lavallée lesion. When suspected, careful evaluation and judgment is essential to avoid surgical site infection. The only shortcoming of the GWP is that it requires that the patient is in the prone position, which seems more disadvantageous for reduction than the supine position. However, if necessary, a Schanz screw from the PSIS can help to reduce the displaced ilia [23].

There are several limitations that could affect the results in the current study. First, this study is a retrospective analysis from our institution, and a control group was not included. Because of the low incidence of unstable pelvic ring injuries in our country, a randomized controlled trial is difficult to perform even at a level 1 trauma center. Further controlled clinical study with a sufficient number of patients is required. Second, selection bias in the outcome measurement may occur in our patient cohort because vertically displaced or bilateral severe sacroiliac joint injury patients who may have a poor outcome were excluded in the current study. Third, radiographic assessment of vertical deformity is missing in the current study, although a rotational deformity parameter was measured on the axial CT slice. Because GWP was not actually applied to severe vertical deformity fractures, it was difficult to precisely measure the difference between pre- and post-operative displacement.

## Conclusions

The GWP represents a simple, safe, minimally invasive procedure with fewer complications and less radiation exposure. It can be used for many fracture types, achieve reliable stability of the posterior pelvic construct, and accelerate rehabilitation. Further study is necessary to investigate the long-term outcomes and compare them to those of alternative surgical techniques.

## Data Availability

The datasets of this study are available from the corresponding author upon reasonable request.

## Abbreviations

GWP: Gull wing plate
CT: Computed tomography
FIM: Functional independence measure
PSIS: Posterior superior iliac spine
S1: The first sacral vertebra
PVM: Paravertebral muscle
AIIS: Anterior inferior iliac spine
POD: Post-operative days
ISS: Injury severity score
AO/OTA: AO Foundation/Orthopaedic Trauma Association
TAE: Transcatheter arterial embolization
